# BMI Is a Poor Predictor of Nutritional Status in Disabled Children. What Is the Most Recommended Method for Body Composition Assessment in This Pediatric Population?

**DOI:** 10.3389/fped.2019.00226

**Published:** 2019-06-06

**Authors:** Valeria Calcaterra, Gloria Pelizzo, Hellas Cena

**Affiliations:** ^1^Pediatric and Adolescent Unit, Department of Internal Medicine, University of Pavia, Pavia, Italy; ^2^Pediatric Unit, Department of Maternal and Children's Health, Fondazione IRCCS Policlinico San Matteo, Pavia, Italy; ^3^Pediatric Surgery Department, Children's Hospital G. Di Cristina, ARNAS Civico-Di Cristina-Benfratelli, Palermo, Italy; ^4^Laboratory of Dietetics and Clinical Nutrition, Department of Public Health, Neurosciences, Experimental and Forensic Medicine, University of Pavia, Pavia, Italy; ^5^Clinical Nutrition and Dietetics Service, Unit of Internal Medicine and Endocrinology, ICS Maugeri IRCCS, Pavia, Italy

**Keywords:** BMI, nutrition, children, disability, body composition

Evidence shows the importance of nutritional assessment in clinical medicine especially in target groups at risk for nutrition imbalances, including children with disabilities in whom the cause of poor nutritional status can be multifactorial, such as decreased nutrient intake, oral motor dysfunction, increased nutrient losses, abnormal energy expenditure, and physical exertion (1).

The body mass index (BMI), calculated as weight in kilograms divided by height in meters squared, is widely used for relating weight to height, defining body size, indicating nutritional status (2) and also for assessing the risk for mortality in the different BMI categories (3–6). Even though the relationship between BMI and mortality remains controversial, substantial data in the published literature have found a J-shaped association between BMI and overall mortality: excessively high or low body mass indexes have been linked to an increased risk of premature mortality (6). As reported by Bhaskaran et al. (4) for mental and behavioral, neurological causes, only a lower BMI was associated with increased mortality risk.

In early infancy (7) as well as in population-based studies (2) by virtue of its wide acceptance in defining specific categories of body mass as a health issue, BMI is considered an adequate indicator of body composition; however in childhood, adolescence and clinical targets it is acknowledged that BMI is a rather poor indicator of body composition, it does not differentiate between lean body mass and body fat mass, which are very important markers of malnutrition impacting disease outcome (2).

Adequate feeding regimens provided by parenteral and enteral therapeutic measures in this target group should be monitored by continuous nutritional assessment, using body composition methods that are far more reliable than BMI; this information is essential for all primary care practitioners.

Recently, in a study by Whitney et al. (8), the authors specified that the prevalence of overweight/obesity status using BMI may be underestimated in children with cerebral palsy (CP). These findings highlight the role of an accurate body composition assessment to estimate fat and free fat mass and the authors recommend statistical models to improve assessment. We agree with the authors that standard anthropometric measures (i.e., height, weight, and BMI) are poor predictors of body composition and hydration status in this population. Furthermore, the measures of height or recumbent length are not always attainable in these children.

Despite the fact that Stevenson's equation can be used to estimate height in non-ambulatory children with CP, in several cases, for example those with skeletal deformities, estimation of height remains inaccurate thereby limiting BMI reliability.

We would also like to point out the lack of a proper classification for underweight patients when using BMI thresholds. Children and young adults with severe disabilities are particularly vulnerable to malnutrition and an underweight status can be indicative of poor nutritional status, affecting the patient's general health (1, 9)

BMI alone does not capture the severity of under nutrition since it is not a good indicator of body composition, nor is it a diagnostic indicator of body fat mass (FM) and/or body fat free mass (FFM) shifts. Decreased FFM as well as increased FM in children with CP may lead to an increased risk of adverse clinical outcomes, including greater mortality rates, Wells, (10) particularly if dehydration is present (11)

Different methods, including dual-energy X-ray absorptiometry (DXA) (8) or indirect methods such as doubly-labeled water (DLW) (12) and bioelectrical impedance analysis (BIA) (9, 12) have been described to assess body composition in the disabled pediatric population, however, the most appropriate method remains to be defined.

Whitney et al. (8) proposed the use of whole-body DXA for this purpose. This method suffers from a plethora of pitfalls that should be considered such as cost and accessibility, time-consumption, exposure to ionizing radiation during measurement, inapplicability in severe forms of disability, as well as being impractical for continued monitoring. Moreover, soft tissue data accuracy has received insufficient attention (13) and poor detection of intra individual changes poses severe limitations. Thus, although DXA studies have increasingly contributed to the evidence base for clinical pediatrics, DXA does not represent a reference method in clinical practice, with the exception of monitoring pediatric bone status (14).

Polfuss et al. (15), calculated body fat% and fat-free mass (FFM) based on total body water measurements from DLW in children with developmental disabilities. This method is considered the gold standard to optimize energy expenditures and to access real body composition data. However, in the severely disabled, blood, urine, or saliva collection can be difficult as well as control of water ingestion. Moreover, the high cost and the high level of competence required for the production of valid data must be considered (16).

Even though indirect methods tend to have larger predictive errors than direct methods and are affected by sample specificity and disease conditions, in our opinion, BIA which is fast, simple, valid and reliable for body composition assessment in “frail” (17) disabled children offers the potential for both non-invasive single assessment and serial monitoring. Whereas, more sophisticated approaches to BIA, such as segmental measurements, improve accuracy and may better describe the direction of changes in FFM and body hydration (13, 18).

As reported by Duren et al. (19), it is also mandatory to remember that all body composition methods are based on assumptions regarding the density of body tissues, concentration of water and electrolytes, and/or biological interrelationships between body components and body tissues and their distributions among healthy individuals. In disabled children, as in other chronic conditions, metabolic and hormonal problems with associated comorbid conditions, may alter the underlying assumptions, interrelationships and validity of body composition methods. Therefore, multiple assessment techniques used in combination (18) may provide the pediatrician with greater accuracy in examining and characterizing body composition also in this population. DXA statistical models should be tested using body composition data obtained with BIA in order to implement clinical practice with simple and affordable tools. Additionally in our opinion, the accuracy of the method cannot be restricted to statistical analysis; commonly used statistical methods can give optimistic or unreliable estimations of accuracy if results are not placed in proper context.

Additional studies on improved body composition assessment in disabled children should help to improve their living conditions and protect these “frail” subjects. This population (similar to the elderly population) is in a state of vulnerability, including poor resolution of homoeostasis after stressor events, which may lead to consequential cumulative decline in many physiological systems and ultimately adverse health outcomes (17).

## Author Contributions

VC, GP, and HC drafted the manuscript and approved the final version.

### Conflict of Interest Statement

The authors declare that the research was conducted in the absence of any commercial or financial relationships that could be construed as a potential conflict of interest.
